# Potential Impact of Omega 6/3 Ratio and CD68+ Macrophage Infiltration on Survival in NSCLC Patients Undergoing Pulmonary Resection

**DOI:** 10.3390/curroncol31090377

**Published:** 2024-08-30

**Authors:** Carlos Déniz, Camilo Moreno, Iván Macía, Francisco Rivas, Anna Ureña, Anna Muñoz, Ines Serratosa, Samantha Aso, Marta García, Cristina Masuet-Aumatell, Ignacio Escobar, Ricard Ramos

**Affiliations:** 1 Thoracic Surgery Department, Hospital Universitari de Bellvitge, Carrer de Feixa Llarga s/n, L’Hospitalet de Llobregat, 08907 Barcelona, Spainimacia@bellvitgehospital.cat (I.M.); frivas@bellvitgehospital.cat (F.R.); amunozf@bellvitgehospital.cat (A.M.); iserratosa@bellvitgehospital.cat (I.S.); mgarciami@bellvitgehospital.cat (M.G.); iescobar@bellvitgehospital.cat (I.E.); 2 Thoracic Surgery Department, Hospital Clínic i Provincial, Carrer de Villaroel 170, 08006 Barcelona, Spain; aurenal@clinic.cat (A.U.); rramosi@clinic.cat (R.R.); 3 Institut d‘Investigacions Biomèdiques August Pi i Sunyer-IDIBAPS, 08036 Barcelona, Spain; 4 Pneumology Department, Hospital Universitari de Bellvitge, Carrer de Feixa Llarga s/n, L’Hospitalet de Llobregat, 08907 Barcelona, Spain; saso@bellvitgehospital.cat; 5 Preventive Medicine and Public Health Department, Hospital Universitari de Bellvitge, Carrer de Feixa Llarga s/n, L’Hospitalet de Llobregat, 08907 Barcelona, Spain

**Keywords:** thoracic surgery, lung cancer, omega fatty acids, omega-6/omega-3 fatty acid ratio, inflammation, CD68

## Abstract

**Background:** Lung cancer remains the leading cause of cancer-related mortality worldwide with non-small cell lung cancer (NSCLC) accounting for the majority of cases. The stage of detection significantly influences survival rates with early-stage diagnosis offering the best prognosis. This study investigates the prognostic impact of the omega-6/omega-3 ratio and tumor infiltration by CD8+ lymphocytes and CD68+ macrophages on overall survival (OS) and disease-free survival (DFS) in NSCLC patients undergoing pulmonary resection. **Methods**: We conducted a retrospective analysis of 53 patients with early-stage NSCLC who underwent pulmonary resection between September 2017 and January 2020. The omega-6/omega-3 ratio was quantified using gas chromatography and spectrometry. Tumor infiltration by CD8 and CD68 was assessed through immunohistochemistry. Survival outcomes were evaluated using Kaplan-Meier and Cox regression analyses. **Results**: An increased omega-6/omega-3 ratio and higher CD68+ macrophage infiltration were associated with a trend towards worse OS and DFS in NSCLC patients, though these results did not reach statistical significance. CD8+ T-cell infiltration was associated with improved survival outcomes, confirming its role as a favorable prognostic marker. Comparative analysis with existing datasets revealed similar demographic and clinical characteristics, reinforcing the generalizability of our findings. **Conclusions**: The omega-6/omega-3 ratio and CD68+ macrophage infiltration serve as important factors potentially influencing prognosis in NSCLC patients undergoing pulmonary resection. These findings highlight the need for further research to refine the prognostic utility of these biomarkers and to explore therapeutic strategies targeting inflammation and immune cell infiltration.

## 1. Introduction

Lung cancer remains the leading cause of cancer-related mortality worldwide despite advances in early detection through screening programs [1]. Non-small cell lung cancer (NSCLC) constitutes approximately 85% of all lung cancer cases [2]. The five-year survival rate for patients with NSCLC is significantly influenced by the stage at which the disease is diagnosed, with early-stage detection offering the best prognosis [3].

Omega-6 and omega-3 polyunsaturated fatty acids (PUFAs) play a crucial role in modulating inflammation, which is a key factor in cancer progression [4]. An imbalance in the omega-6/omega-3 ratio has been associated with worse prognostic outcomes in various cancers, including NSCLC [5]. Previous research suggests that a high omega-6/omega-3 ratio may enhance inflammatory pathways, thereby contributing to tumor growth and metastasis in NSCLC patients [6,7]. This hypothesis is supported by findings indicating that a higher omega-6/omega-3 ratio is linked to the promotion of pro-inflammatory eicosanoids, which are involved in tumorigenesis and cancer progression [8].

Tumor infiltration by immune cells, particularly CD8+ lymphocytes and CD68+ macrophages, has been recognized as a significant prognostic factor in lung cancer [9,10]. Studies have shown that CD8+ T-cell infiltration is associated with improved overall survival (OS) and disease-free survival (DFS) across different stages of lung cancer, highlighting their role as a favorable prognostic marker [11]. CD68+ macrophages, often referred to as tumor-associated macrophages (TAMs), can influence the tumor microenvironment and impact cancer progression. TAMs can promote tumor growth and metastasis through the release of growth factors and cytokines, making them a target of interest for prognostic evaluation [12,13].

Given the potential prognostic significance of the omega-6/omega-3 ratio and immune cell infiltration, this study aims to evaluate their combined impact on OS and DFS in patients with NSCLC undergoing pulmonary resection. By understanding these interactions, we hope to uncover novel insights into their influence on patient outcomes.

## 2. Materials and Methods

### 2.1. Study Population

A retrospective study was conducted on a cohort of 53 patients treated with radical intent through pulmonary resection between September 2017 and January 2020. This cohort has not been previously reported in similar studies. However, previous analyses of lung cancer datasets have shown comparable demographic and clinical characteristics, such as age, gender distribution, and histological subtypes. All patients included were diagnosed with early-stage NSCLC and were suitable candidates for anatomical lung resection with systematic lymph node dissection. This study was approved by the Clinical Research Ethics Committee of our hospital (approval number: XYZ123), and all participants provided informed consent.

### 2.2. Exclusion Criteria

Patients with a history of systemic inflammatory disease, active concomitant infection, neoadjuvant treatment, patients lost to follow-up, and those lacking preoperative studies were excluded. Specific systemic inflammatory disorders included were arthritis, lupus, and Crohn’s disease.

### 2.3. Preoperative Assessment

All patients underwent a standardized preoperative assessment, including physical examination, blood tests, bronchoscopy, pulmonary function testing, CT, and PET-CT.

### 2.4. Blood Quantification Method for Ω6/Ω3 Ratio

Blood samples were collected using EDTA as an anticoagulant after anesthesia induction and before starting surgery. The blood was allowed to clot for a minimum of 30 min, then centrifuged at 700× *g* within the first hour of extraction. The samples were aliquoted and frozen at −70/−80 °C until analysis. The ω6/ω3 ratio was quantified using gas chromatography (model: Agilent 7890B (Agilent, Barcelona, Spain)) and spectrometry (model: Agilent 5977A) conducted by the Scientific Service of the University of Barcelona. The optimal cut-off point for the omega-6/omega-3 ratio was determined using the Youden index, maximizing the sum of sensitivity and specificity for predicting survival outcomes.

### 2.5. Immunohistochemical Analysis

Paraffin-embedded tumor sections were prepared and stained with antibodies against CD8 T cells and CD68 macrophages. The immunohistochemical analysis was conducted by an experienced pathologist, who evaluated the proportion of CD68 and CD8 positive cells. Five high-power fields were assessed per slide, and the number of lymphocytes and macrophages was recorded in each field.

### 2.6. Statistical Analysis

Descriptive analysis was conducted using frequencies and percentages for qualitative variables and mean and standard deviation for quantitative variables if normally distributed. The omega-6/omega-3 ratio variable was dichotomized using the Youden index. Kaplan-Meier survival curves and Cox regression were used to evaluate associations between variables and survival outcomes. Both bivariate and multivariate analyses were conducted using SPSS software version 16.0.

## 3. Results

### 3.1. Patient Characteristics

The study included 53 patients, with a mean age of 63.9 ± 10.3 years. Among them, 73.6% were male and 26.4% were female. The most common histology was adenocarcinoma, representing 67.9% of the cases, followed by squamous cell carcinoma at 15.1%. These demographic and clinical characteristics are consistent with those reported in other lung cancer studies, supporting the generalizability of our findings (Table 1).

### 3.2. Preoperative Nutritional and Inflammatory Study

Preoperative nutritional and inflammatory parameters were assessed for all patients. The mean omega-6/omega-3 ratio was 17.39 ± 9.45, with a range of 5.7 to 34.1. The cut-off point of 21 for the omega-6/omega-3 ratio was determined using the Youden index, which maximizes the sum of sensitivity and specificity for predicting survival outcomes. Additional preoperative measures, including Body Mass Index (BMI), Prognostic Nutritional Index (PNI), and various inflammatory markers, are summarized in Table 2.

### 3.3. Tumor Infiltration by Immune Cells

**Figure 1:**
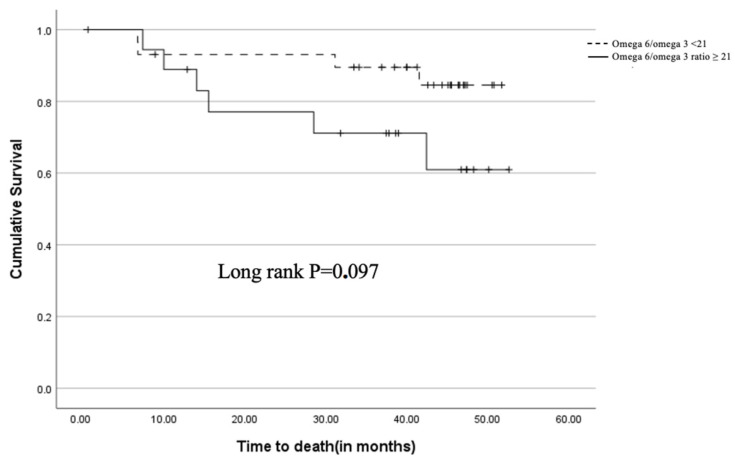
Kaplan-Meier survival for overall survival based on the omega 6/omega 3 ratio groups (Omega 6/omega 3 < 21; Omega 6/omega 3 ratio ≥ 21).

The immunohistochemical analysis, conducted by an experienced pathologist, revealed a mean CD8+ T-cell count of 106.9 ± 65.47 cells per high-power field (HPF) and a mean CD68+ macrophage count of 115.19 ± 62.96 cells per HPF (Figure 1). Although the images for CD68 and CD8 staining were not available for inclusion, the analysis was consistent across the samples evaluated.

### 3.4. Survival Analysis

**Figure 2:**
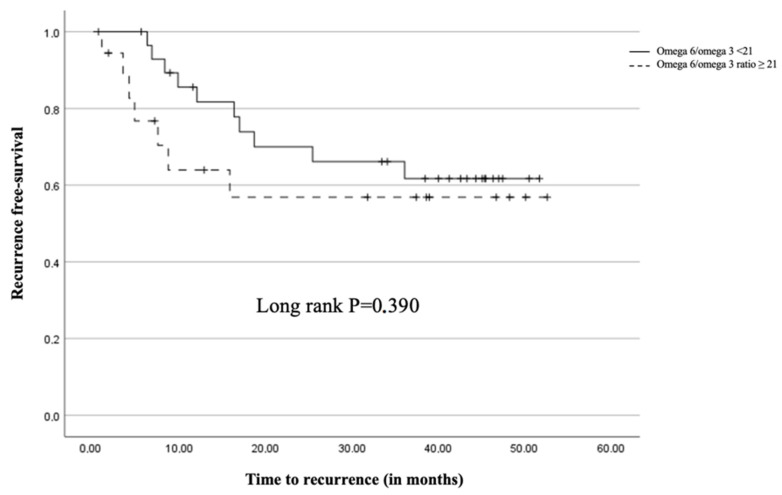
Kaplan-Meier survival for recurrence free-survival based on the omega 6/omega 3 ratio groups (Omega 6/omega 3 < 21; Omega 6/omega 3 ratio ≥ 21).

Survival outcomes were analyzed using Kaplan-Meier curves and Cox regression models. Patients with an omega-6/omega-3 ratio ≥ 21 showed a trend towards worse overall survival (OS) and disease-free survival (DFS) compared to those with a ratio < 21. However, this trend did not reach statistical significance (OS: *p* = 0.097, DFS: *p* = 0.390) (Figure 2).

Increased infiltration by CD68+ macrophages was significantly associated with poorer OS in the multivariate analysis (*p* = 0.018), while infiltration by CD8+ T-cells was associated with improved survival outcomes (Table 3). These findings suggest a potential role for these immune markers in influencing patient prognosis.

### 3.5. Recurrence and Mortality

At the end of the follow-up period, 18 patients (33.9%) experienced recurrence, and 10 patients (18.9%) had passed away. The recurrence rate was higher in patients with an omega-6/omega-3 ratio ≥ 21 (36.8% vs. 33.3% in those with a ratio < 21), although this difference was not statistically significant.

### 3.6. Multivariate Analysis

Multivariate analysis confirmed a significant association between worse overall survival and several factors: CD68+ macrophage infiltration (*p* = 0.018), age (*p* = 0.016), male gender (*p* = 0.043), Thoracoscore index (*p* = 0.018), omega-6/omega-3 ratio ≥ 21 (*p* = 0.020), adenocarcinoma histology (*p* = 0.027), and pathological staging (*p* = 0.027) (Table 3).

## 4. Discussion

The findings of this study suggest that both the omega-6/omega-3 ratio and CD68+ macrophage infiltration play important roles in the prognosis of patients with non-small cell lung cancer (NSCLC) undergoing pulmonary resection. While the trend towards worse overall survival (OS) and disease-free survival (DFS) associated with a higher omega-6/omega-3 ratio did not reach statistical significance, the data indicate a potential link that warrants further investigation.

Omega-3 fatty acids have been widely studied for their anti-inflammatory properties and their role in modulating immune responses, particularly in cancer [14]. An imbalance in the omega-6/omega-3 ratio, characterized by an excess of omega-6 fatty acids, has been linked to increased production of pro-inflammatory eicosanoids, which can promote tumorigenesis and cancer progression [15]. The chosen cut-off point for this ratio highlights its potential importance in patient prognosis.

The role of tumor-infiltrating immune cells, particularly CD8+ T cells and CD68+ macrophages, has been established as a significant factor in the prognosis of various cancers, including NSCLC [16,17]. In our study, CD68+ macrophage infiltration was significantly associated with poorer survival outcomes, supporting the notion that tumor-associated macrophages (TAMs) contribute to a more aggressive tumor phenotype. This finding aligns with previous studies that have shown a correlation between high TAM infiltration and reduced survival in NSCLC [18].

On the other hand, CD8+ T-cell infiltration was associated with improved survival, which is consistent with their role as cytotoxic lymphocytes that target and destroy cancer cells [19]. These results reinforce the importance of the tumor microenvironment in influencing the progression of NSCLC and suggest that therapies aimed at modulating the immune response could be beneficial.

It is important to note that the lack of statistical significance in the survival analysis related to the omega-6/omega-3 ratio may be due to the relatively small sample size, which limits the power of the study. Future studies with larger cohorts are necessary to validate these findings and explore the underlying mechanisms that link fatty acid balance to cancer progression.

Additionally, while the current study focused on the CD68+ macrophage population, further research should include specific markers for different subsets of tumor-associated macrophages, such as M1 and M2 macrophages, to better understand their distinct roles in tumor biology [20]. The distinction between these subtypes could provide more targeted insights into how macrophages influence tumor progression and patient outcomes.

The implications of these findings extend beyond prognosis, as they may inform the development of new therapeutic strategies. For example, targeting the omega-6/omega-3 balance through dietary interventions or pharmacological means could potentially modulate the inflammatory environment in tumors, thereby improving patient outcomes [21]. Similarly, therapies that alter macrophage polarization from a pro-tumorigenic to an anti-tumorigenic state could also prove beneficial in the treatment of NSCLC [22].

## 5. Study Limitations

This study has several limitations that should be considered when interpreting the results. The retrospective design and relatively small sample size limit the generalizability of the findings. Additionally, the assessment of CD68+ and CD8+ cell infiltration was based on immunohistochemical analysis, which, while reliable, is subject to observer bias and variability. Future studies should aim to include larger, prospectively collected cohorts and utilize more advanced techniques for assessing immune cell infiltration and fatty acid composition.

In conclusion, while the omega-6/omega-3 ratio and CD68+ macrophage infiltration were associated with trends towards poorer survival in NSCLC patients, further research is needed to confirm these findings and explore their potential as therapeutic targets. The role of the tumor microenvironment, particularly the balance of immune cell populations and fatty acids, remains a critical area of study in the quest to improve outcomes for patients with NSCLC.

## 6. Conclusions

The omega-6/omega-3 ratio and CD68+ macrophage infiltration may serve as important prognostic markers in NSCLC, with further research needed to validate these findings and explore their therapeutic potential.

## Figures and Tables

**Table 1:** Clinicopathological and surgical characteristics of the patients.

Variable	N (%)
Age	63.9 (±10.3) *
Male sex	39 (73.6%)
Active smoker	15 (28.3%)
Diabetes	16 (30.2%)
COPD	38 (71.7%)
Ischemic Heart Disease	6 (11.3%)
With previous pulmonary neoplasia	1 (1.9%)
Without previous pulmonary neoplasia	19 (35.8%)
Cerebral vascular accident (CVA)	1 (1.9%)
Peripheral vasculopathy	2 (3.8%)
Previous cardiac surgery	1 (1.9%)
Nephropathy	3 (5.7%)
Dyslipidemia	20 (37.7%)
Hypertension	27 (50.9%)
ASA	3.95 (±0.99)
1	6 (11.3%)
2	35 (66%)
>3	12 (22.6%)
Maxime Expiratory Volume in the first second (FEV)	100.44 (±94.95) *
Thoracoscore Index	
Histology	
Adenocarcinoma	36 (67.9%)
Squamous cell carcinoma	8 (15.1%)
Carcinoid Tumour	2 (3.8%)
Large Cell	4 (7.5%)
Metastasis CCR	3 (5.7%)
Pathological staging	
Ia1	1 (1.9%)
Ia2	9 (17.3%)
Ia3	4 (7.7%)
Ib	13 (25%)
IIa	1 (1.9%)
IIb	11 (21.2%)
IIIa	6 (11.5%)
IIIb	2 (3.8%)
Surgery	
Lobectomy	46 (86.8%)
Wedge	7 (13.2%)
Approach	
Thoracotomy	27 (50.9%)
Thoracoscopy/VATS	26 (49.1%)

* IQR: Median ± Interquartile Range.

**Table 2:** Preoperative Nutritional and Inflammatory Study.

Variable	Mean or Median (SD or IQR)
Height	1.66 (±0.09)
weight	75.61 (±14.22)
BCMI (*Body Cell Mass Index*)	11 (±2.33)
BCM (*Body Composition Monitor*) (kg)	30.29 (±8.01)
FFM (*Fat Free Mass*) (kg)	54.6 (±9.92)
FM (*Fat Mass*) (kg)	19.52 (±8.92)
Adherence to the Mediterranean diet Test	40 (±58.82)
BMI (Body Mass Index)	27.06 (±4.96)
IPN (Prognostic Nutritional Index)	47.1 (±5.85)
Preoperative Plasmatic Test	
Albumin	39.63 (±3.75) *
Prealbumin	225.05 (±67.69) *
Cholesterol	34.64 (117.91)
Vitamin D	35.78 (±21.05) *
Fatty acids	
Ratio Omega 6/3	17.39 (±9.45)
EPA	0.36 (±0.29)
DHA	1.23 (±0.71)
Linolenic Acid	0.41 (±0.27)
ARA	6.33 (±1.90)
Linoleic Acid	24.07 (±7.40)
Inflammatory Parameters	
Neutrophils	4720.44 (±2398.10)
Lymphocytes	1561.03 (±779.70)
Platelets	205,177.94 (±79,743.10)
Monocytes	525.59 (±206.60)
Ratio Neutrophils/Lymphocytes	3.91 (±3.64)
Ratio Platelets/Lymphocytes	152.32 (±70.36)
Ratio Lymphocytes/Monocytes	3.3 (±1.64)
Transferrin	41.66 (±58.30)
PCR	9.04 (±16.22)
Fibrinogen	4.05 (±51.63) *
SII (*Systemic Inmune-inflamation Index*)	12,162.21 (±581,087.45)
Immunohistochemical analysis	
CD8	106.9 (±65.47)
CD68	115.19 (±62.9)

* IQR: Median ± Interquartile Range.

**Table 3:** Significant Associations with Overall Survival: Multivariate Analysis Findings.

	Hazard Ratio	95.0% CI		Sig.
		Upper	Lower	
CD68	1.376	1.056	1.793	0.018
Age	0.173	0.042	0.716	0.016
Male	0.001	0.001	0.697	0.043
Thoracoscore Index	0.001	0.001	0.004	0.018
Omega 6/Omega 3 ratio ≥ 21	0.001	0.001	0.018	0.02
adenocarcinoma histology	0.001	0.001	0.193	0.027
pathological staging	0.001	0.001	0.218	0.027
Lpreoperative MST	0.001	0.001	0.001	0.015

## Data Availability

The original contributions presented in the study are included in the article, further inquiries can be directed to the corresponding author.
